# Developing an Instrument to Measure Public Health Nurses’ Competence Related to Breastfeeding Beyond 12 Months

**DOI:** 10.1177/08903344241254343

**Published:** 2024-05-29

**Authors:** Niina Pöyhönen, Oona Ojantausta, Marja Kaunonen, Katri Vehviläinen-Julkunen, Riikka Ikonen

**Affiliations:** 1 Department of Nursing Science, Faculty of Health Sciences, University of Eastern Finland, Kuopio, Finland; 2 Faculty of Social Sciences, Health Sciences, Tampere University, Tampere, Finland; 3 Pirkanmaa Wellbeing Services, General Administration, Tampere, Finland; 4 North Savo Wellbeing Services County, Kuopio, Finland

**Keywords:** attitude of health personnel, breastfeeding, breastfeeding knowledge, clinical competence, health personnel, instrument development, lactation prolonged

## Abstract

**Background:**

Health professionals need adequate competence to support breastfeeding beyond infancy. There is no established instrument to measure health professionals’ competence regarding long-term breastfeeding. To respond to this shortcoming, the Long-Term Breastfeeding Competence Scale (LBCS) was developed.

**Research Aim:**

To develop and pilot an instrument that measures public health nurses’ competence related to breastfeeding beyond 12 months in order to provide adequate breastfeeding counseling for families.

**Methods:**

This study was conducted as a cross-sectional online survey on public health nurses working in maternity and/or child health clinics. The relevance and clarity of the LBCS were assessed by an expert panel (*N* = 6). Public health nurses (*N* = 197) completed the LBCS, which consisted of a knowledge and skills dimension and an attitude dimension. Descriptive statistics were used to describe the characteristics of the study sample. The conceptual validity of the knowledge and skills dimension was assessed using the dichotomous Rasch analysis, and attitude dimension using the exploratory factor analysis. Internal consistency was evaluated using Cronbach’s alpha. The distribution of the items was summarized by descriptive statistics.

**Results:**

According to expert panel evaluations, the LBCS was found to meet the requirements for relevance and clarity (S-CVI 0.90). The internal consistency of the instrument was at a good level (*α* = 0.796) and met the requirements set for a new instrument.

**Conclusion:**

The LBCS is appropriate to determine public health nurses’ competence related to breastfeeding beyond 12 months. The LBCS can be used to identify the need for education concerning breastfeeding beyond 12 months.

## Key Messages

- There is no previously established instrument to measure public health nurses’ competence (namely knowledge, skills, and attitudes) regarding breastfeeding beyond 12 months.
- To provide adequate breastfeeding counseling for families, public health nurses need a sufficient level of competence regarding long-term breastfeeding.
- The Long-Term Breastfeeding Competence Scale (LBCS) is an applicable instrument for examining public health nurses’ competence regarding breastfeeding beyond 12 months.

## Background

The World Health Organization (WHO) recommends continuing breastfeeding until at least 2 years of age. (Victora et al., 2016; WHO, 2023) Despite global recommendations, there is a worldwide lack of adequate professional education regarding breastfeeding beyond infancy and little is known about the specific counseling content for long-term breastfeeding (Guevara, 2017, Tchaconas et al., 2018). Supporting breastfeeding beyond 12 months requires different approaches compared to supporting breastfeeding during infancy (Tchaconas et al., 2018), as breastfeeding after the baby’s first year of life serves purposes beyond nutrition, for example, soothing, calming, and aiding the child’s sleep (Thompson et al., 2020). This understanding is often lacking in healthcare settings (Blixt et al., 2019; Dowling & Brown, 2013).

Breastfeeding counseling is not only giving advice to families but also supporting mothers’ self-esteem and self-confidence (de Almeida et al., 2015). Health professionals have a significant influence on mothers through their advice and expressions (Corr et al., 2015), and breastfeeding counseling is influenced not only by knowledge but also by the professional’s personal characteristics (de Almeida et al., 2015). Mothers face negative attitudes towards long-term breastfeeding from health professionals and society (Cockerham-Colas et al., 2012; Dowling & Brown, 2013; Tomori et al., 2016), and may feel pressure to stop breastfeeding due to lack of support (Dowling & Brown, 2013). Health professionals should provide evidence-based, sensitive, and individualized breastfeeding support for mothers and families (Blixt et al., 2019; Dowling & Brown, 2013).

Health professionals should have adequate clinical competence to normalize and support long-term breastfeeding (Dowling & Brown, 2013; Zhuang et al., 2020). In nursing science, clinical competence has been defined as a combination of knowledge, skills, and attitudes (Garside & Nhemachena, 2013; Nabizadeh-Gharghozar et al., 2021; Valizadeh et al., 2019; Nascimento et al., 2021; Vihelä et al., 2020). The United States Breastfeeding Committee (USBC, 2010) defines breastfeeding core competencies as the minimum knowledge, skills, and attitudes that all health professionals should possess to protect, promote, and support breastfeeding.

Previously published breastfeeding competence scales for health professionals mainly focus on supporting breastfeeding in infancy. When the research subject has been breastfeeding beyond infancy, the focus has typically been on health professionals’ attitudes. To date, there is no established instrument focusing on health professionals’ competence related to breastfeeding beyond 12 months. In this study, an existing instrument evaluating health professionals’ knowledge and attitudes towards long-term breastfeeding, the Extended Breastfeeding Knowledge and Attitudes tool (Cockerham-Colas et al., 2012), was further developed to cover competence in its entirety. The aim of this study was to develop and pilot an instrument that measures public health nurses’ competence related to breastfeeding beyond 12 months, in order to provide adequate breastfeeding counseling for families.

## Methods

### Research Design

This study was conducted as a cross-sectional online survey of public health nurses working in maternity and/or child health clinics. An online questionnaire was used to reach the members of the target group in any location in Finland. An affirmative statement for the research project was obtained from the Ethics Committee of the Tampere Region, Finland on May 9, 2022 (statement nr. 51/2022)

### Setting and Relevant Context

Finnish public maternity and child health clinics lay the foundation for the monitoring of children’s development and providing preventive health care for families. Public health nurses working in maternity and child health clinics support families during pregnancy and within the first 7 years of children’s lives with regular appointments (Ministry of Social Affairs and Health, 2023). Services are free of charge for the clients and 99.6% of Finnish children are covered by the system (Finnish Institute for Health and Welfare, 2023). A public health nurse qualification is mandatory to be able to work in a child health care clinic. The implementation of breastfeeding counseling in maternity and child health clinics is based on national guidelines (Hakulinen et al., 2023). In all, 97% of the Finnish public health nurses working in maternity and child health clinics have completed the WHO 20-hour Breastfeeding Counselor Course (Ikonen et al. 2020). In Finland, public health nurses are the primary professionals to support families in breastfeeding during pregnancy and childhood. Public health nurses may hold additional degrees, for example International Board Certified Lactation Consultant (IBCLC) or midwife.

In Finland, breastfeeding is recommended for up to 1 year, or longer if the family so desires (Finnish Institute for Health and Welfare, 2019). Breastfeeding rates have improved in Finland during the previous decade and more than half (58%) of babies nearing the age of 1 year are still breastfed (Ikonen et al., 2020). National guidelines for breastfeeding support end at the 1-year milestone, after which the counseling is focused on a transition to cow’s milk or a plant-based option (Finnish Institute for Health and Welfare, 2022).

#### Sample

The target population for this study was public health nurses working in maternity and child health clinics. To meet the inclusion criteria, participants had to currently work in maternity and/or child health clinics, and they also had to be qualified public health nurses. Participants were recruited through public health nurses’ labor unions via email, and they did not receive any compensation for their participation. The language of the questionnaire was Finnish. Based on the sample-to-item ratio 5-to-1 (Memon et al., 2020), a sufficient sample size was estimated to be 175, as the number of items in the instrument was 35. It is also estimated that 100–200 respondents guarantee the variability required by the evaluation methods chosen for the instrument (Chen et al., 2014; Hertzog, 2008). The sample size was determined based on these requirements, as information on the exact size of the target population was not available. Participants who only responded to the background items, were excluded. Based on this, 28 of the 225 participants were rejected. The final sample size was *N* = 197.

### Measurement

This study piloted an instrument, the Long-Term Breastfeeding Competence Scale (LBCS) which measures the knowledge, skills and attitudes of health professionals. The LBCS was based on the Extended Breastfeeding Knowledge and Attitudes tool (Cockerham-Colas et al., 2012), which consisted of five knowledge items and 20 attitude items. Permission to translate and modify the instrument was received from the developer. The original English instrument was first translated into Finnish, then back-translated with a professional translator, and the versions were compared (Maneesriwongul & Dixon, 2004; Nilsson Kajermo et al., 2013). The translated instrument was complemented with six new items related to knowledge and skills that were operationalized based on a systematic review of health professionals’ competencies regarding breastfeeding beyond 12 months. Four of the original knowledge items were adaptable to the Finnish context and were included in the LBCS.

#### Development of the instrument based on the expert panel evaluations

A six-member expert panel was assembled to assess content validity and face validity and to inform any necessary modifications required to ensure the instrument’s clarity and feasibility. The panel members were public health nurses, health care managers, experts in breastfeeding counseling, and breastfeeding researchers in different disciplines. The members were invited to the panel by the research group as the researchers had a broad understanding of breastfeeding expertise in Finland. In such a small language area, practically all long-term breastfeeding experts were involved. Therefore, it was possible to form a versatile and professional expert panel, and to minimize any bias. The expert panel members did not have any conflicts of interest and did not receive any compensation for their participation in the evaluations. The experts evaluated relevance and clarity of each item on a 4-point scale. The content validity index for the items (I-CVI) was calculated using a method in which the number of experts who rated the item as somewhat relevant (3) or very relevant (4) was divided by the total number of experts, and so the value of I-CVI is between 0 and 1. Based on the evaluations, the content validity index for the scale (S-CVI) was determined by calculating the average of the item content validity indexes (I-CVI; DeVon et al., 2007; Zamanzadeh et al., 2015). The experts also had an opportunity to comment on each item and the instrument as a whole, and their suggestions were taken into account in the development of the instrument.

According to the expert panel’s (*N* = 6) evaluations, the LBCS was found to meet the requirements for relevance and clarity. The content validity index of the entire instrument (S-CVI) was 0.90. The item content validity index (I-CVI) was at a satisfactory level (I-CVI 0.83–1.0) for 27 items, and thus these items were found to be relevant. The content validity index for the relevance of three of the statements was 0.66. However, a decision was made to nonetheless include them in the piloting of the instrument, because two of the items were from the original instrument (Cockerham-Colas et al., 2012), and the third was based on international research evidence. The clarity of the instrument was invariably rated good by the experts (I-CVI 0.83–1.0). The experts also provided suggestions regarding the clarity of the language, the cultural appropriateness and the order of the items as well as the technical functionality of the instrument. Based on the experts’ opinions, the order of the knowledge and skills items was modified, and a few items were clarified linguistically. The “1–2-year-old child” and “3–4-year-old child” in the original instrument’s attitude item response alternatives were divided into separate age groups: “1-year-old child,” “2-year-old child,” and “3-year-old or older child.” This change was made to clarify the options and to enable the examination of the attitudes before and after the WHO’s 2-year breastfeeding recommendation cut-off point.

#### Instrument used in the pilot study

The sociodemographic background variables included age, gender, professional education, education related to breastfeeding counseling, work unit, work experience years, and personal parental and breastfeeding background. In addition, the respondents were asked about their participation in breastfeeding education during the previous 5 years, participation in education related to breastfeeding beyond 12 months and experience of the necessity of education about breastfeeding beyond 12 months.

The knowledge and skills dimension contained 10 items related to breastfeeding beyond 12 months. Of those items, four were from the original Extended Breastfeeding Knowledge and Attitudes tool (Cockerham-Colas et al., 2012). The original items considering breastfeeding during pregnancy and mother’s breast cancer risk were included unedited. The items considering breastfeeding statistics and recommendations were modified into the Finnish context. Six new knowledge and skills items were operationalized based on a systematic review (Ojantausta et al., 2023) and study findings previously published in the research project (Ojantausta & Kaunonen, 2021). The new items considered nighttime breastfeeding, financial benefits of breastfeeding, nutritional value of human milk, a child’s obesity risk, attachment relationship between a child and a parent, and initiating a conversation regarding breastfeeding at the standard counseling visit for the 1-year-old child. The items were multiple-choice questions with one correct answer and two incorrect options, or questions with the answer options “true,” “false,” “don’t know.”

The attitude dimension was formed by 25 items. The items were the same as in the original Extended Breastfeeding Knowledge and Attitudes tool, but the number of total items increased as the categories regarding the child’s age (under 1 year old, 1–2 years old, 3–4 years old and, in some items, also 4 years old or older) were modified to prevent the partial overlap of groups. In the LBCS, the categories of the child’s age were “under 1 year old,” “1-year-old,” “2-year-old,” and “3-year-old or older child” in all items. The first attitude item explored the respondent’s opinion about the normality of breastfeeding a child over 1 year old. The other items measured the respondent’s attitudes by age group from the perspectives of acceptability, embarrassment, health effects to mother’s and child’s physical and psychological health and encouragement to stop breastfeeding. The response options for the attitude items were on a 5-point Likert scale (*completely agree, somewhat agree, neither agree nor disagree, somewhat disagree, completely disagree*). The development and the structure of the LBCS are shown in Table 1. The LBCS is included in its entirety in the online Supplemental Material.

**Table 1.** The Development and the Structure of the Long-Term Breastfeeding Competency Scale.

	Items			
Dimension	Pilot Study (*n*)	Validated Instrument (*n*)	Scale	Score
Knowledge and Skills Dimension	10	9	True/False/Don’t know Multiple choice	Correct answer: 1 point Incorrect answer: 0 points
Attitude Dimension	25	22	5-point Likert scale	Negative: 1 point Somewhat negative: 2 points Neutral: 3 points Somewhat positive: 4 points Positive: 5 points

### Data Collection

The request for participation was distributed between April 11–26, 2023, via email to public health nurses (*n* = 10,953) in the membership newsletters of the Finnish public health nurses’ trade unions. Only part of the population of public health nurses who received the email worked in maternity and child health clinics. No information was available on the exact number of public health nurses working in these environments in Finland.

The data were collected using the RedCap online survey tool. Informed consent was obtained on the starting page of the online survey by asking the participants to read the study information and confirm their agreement to participate before continuing to the survey. To guarantee the confidentiality of the participants, no information from which individual respondents could be identified, including IP address, was collected. Survey data were stored on privacy-protected computers secured by password protection.

### Data Analysis

Descriptive key statistics (medians, quartiles, ranges, frequencies, and percentages) were used to analyze the pilot study data. The correlations of the statements were examined with Spearman’s correlation coefficient (DeVon et al., 2007; Hertzog, 2008). In addition, the frequencies and correlation of sum variables and attitude subscales formed by the factor analysis were tested. There was no missing data in the knowledge and skills dimension (*N* = 197), but there were seven participants who did not respond to any of the attitude items and thus were excluded from the analyses regarding attitude dimension (*n* = 190). Imputation of missing values was not done. The SPSS (Version 27) and Jamovi (Version 2.3.26) statistical programs were used for the analysis.

Necessary modifications were made to the data before performing the analysis. The knowledge and skills items were classified as dichotomous variables (1 = *correct answer*, 0 = *incorrect answer* or *don’t know*). The direction of the Likert scales was aligned with the attitude items (1 = *most negative attitude*; 5 = *most positive attitude*). The response option *neither agree nor disagree* was interpreted as a neutral attitude.

A dichotomous Rasch analysis was utilized to evaluate the knowledge and skills dimension of the instrument. Rasch analysis is used to examine the difficulty of the questions and the level of competence of the respondents (*N* = 197) and to assess whether an instrument measures the desired characteristics and knowledge (unidimensionality; Boone, 2016). Instruments’ unidimensionality was examined by principal component analysis of the residuals with the Varimax rotation method. The target result for the explanatory level of the first component was > 50% and, at most, 5% for the following (Stolt et al., 2022). The item fit to the model was assessed with person reliability (> 0.80), local independency evaluated with Q3 coefficient correlations (< 0.3) and MADaQ3 index numbers (target value close to 0), and fitting parameter values based on INFIT and OUTFIT of mean square error and standardized root mean square error (0.6–1.4; Boone, 2016; Christensen et al., 2017; Matheny & Clanton, 2020; Stolt et al., 2022). Items’ difficulty and the respondents’ ability level were examined with Wright’s pattern and descriptive key figures (Boone, 2016; Stolt et al., 2022).

The conceptual validity of the attitude dimension was assessed by exploratory factor analysis. The method is based on the measurement of correlations between variables and an assumption that the cause of the correlation is a common factor. Based on the assumptions, exploratory factor analysis can be used to outline the latent structures of the instrument and divide it into subscales (DeVon et al., 2007; Hertzog, 2008; Lanario et al., 2020). Missing data were not taken into account in the factor analysis, as only complete responses were included (*n* = 177). The suitability of the data for factor analysis was verified by Bartlett’s test of sphericity (*p* < 0.005), Kaiser-Meyer-Olkin test (KMO > 0.80), and communalities (> 0.500; Williams et al., 2010). Factor analysis was carried out using principal component analysis and Varimax as the rotation method (Costello & Osborne, 2005). Factors were formed by examining the loadings between statements and factors (> 0.500). Statements with weak communality or low loading on the factors were removed until there was a factor model that consisted of meaningful factors and explained the variation of the observed variables well (> 50%–60%; Williams et al., 2010).

The internal consistency of the instrument was tested using Cronbach’s alpha coefficient (DeVon et al., 2007; Hertzog, 2008). The Cronbach’s alpha value required for the new instrument is recommended to be > 0.70. (Bujang et al., 2018). Cronbach’s alpha values were calculated for the entire instrument and subscales.

## Results

### Characteristics of the Sample

There were a total of 197 participants in the survey. The characteristics of the public health nurses are shown in Table 2.

**Table 2.** Sociodemographic Characteristics of the Public Health Nurses (*N* = 197).

Baseline Characteristic	*n* (%)
Age Group	
18–29 years	31 (15.7)
30–39 years	82 (41.6)
40–49 years	49 (24.9)
50 years and older	29 (14.7)
Gender	
Female	195 (99.0)
Rather not say	2 (1.0)
Profession	
Public health nurse	196 (99.5)
In addition, midwife	28 (14.2)
BF Counseling-Related Qualification	
WHO 20 hr BF counselor	123 (62.4)
IBCLC or trainer	11 (5.6)
Working Unit	
Maternity and child health clinic	154 (78.2)
Child health clinic	39 (19.8)
Maternity clinic	4 (2.0)
Years of Work Experience	
< 2 years	31 (15.7)
2–5 years	55 (27.9)
6–10 years	43 (21.8)
11–20 years	49 (24.9)
> 20 years	16 (8.1)
Own Experiences	
Own child/children	172 (87.3)
Own BF experience	170 (86.3)
No child/children	25 (12.7)
BF Education	
Participation in any BF education during previous 5 years	157 (80.7)
Participation in education related to BF beyond 12 months	37 (18.8)
Experience of the need for education related to BF beyond 12 months	146 (74.1)

*Note.* BF = breastfeeding; IBCLC = International Board Certified Lactation Consultant; trainer = Breastfeeding Counselor Trainer (university level additional course for health professionals); WHO = World Health Organization. Missing values: age = 6, profession = 1, BF counseling related qualification = 1, years of work experience = 3.

### Validity and Reliability of the LBCS

The Rasch analysis of the knowledge and skills dimension (*N* = 197) indicated that the respondents’ ability level was good. An item regarding the counseling visits for a 1-year-old child was removed from the analysis to improve the explanatory level of the instrument and Cronbach’s alpha, as it differed from the other items by being significantly easier. The remaining nine items mostly fit the model well (INFIT 0.905–1.140, OUTFIT 0.827–1.623). For one variable, the OUTFIT value exceeded the upper limit of the target value, which may indicate that the respondents had guessed the answer to a challenging question. The local independence of the variables was acceptable (Q3 correlation < 0.3, MADaQ3 0.0738 *p* = 0.016), but the explanatory level of the model remained low (person reliability 0.411). The factorization of the residuals produced a two-factor model with two equally strong factors (explanatory proportions of variance 14.2% and 17.9%). The model therefore did not support the unidimensionality of the instrument’s knowledge and skills dimension. A strong correlation (*p* < 0.001) between the sum of variables formed out of the knowledge and skills dimension and the attitude dimension suggests that knowledge and skills and attitudes are related.

In the exploratory factor analysis, three attitude items concerning breastfeeding during infancy were removed from the instrument. The expert panels’ (*N* = 6) suggestions also supported removal. In these items, the public health nurses’ (*n* = 177) responses were consistently positive, which is why the items did not correlate with the others and undermined the suitability of the correlation matrix for factor analysis. The remaining 22 items of attitude dimension formed a six-factor model. The correlation matrix was well suited for factor analysis (Bartlett’s skewness test *p* < 0.001, KMO 0.807). The commonalities of the statements were at a sufficient level (range 0.561–0.931) and the values obtained for loadings showed a strong commitment to the factors (0.50–0.94). The factor model of six factors explained 74.16% of the variation in the variables, which can be considered a good result.

The internal consistency of the entire instrument and subscales was at a good level, except for the knowledge and skills dimension, and met the requirements set for a new instrument. The Cronbach’s alpha values calculated for the instrument and its subscales, and the factor loadings are shown in Table 3.

**Table 3.** Cronbach’s Alphas and Factor Loadings of the LBCS (*N* = 177).

Instrument, Dimension, or Subscale	Cronbach’s Alpha	Factor Loading					
		1	2	3	4	5	6
Overall instrument	0.80						
Knowledge and skills dimension	0.56						
Attitudes dimension	0.89						
Attitudes Subscale 1: Breastfeeding a 2-year-old or an older child	0.90						
In general, I think breastfeeding a child of 3 years or older would benefit a child’s physical health		**0.89**	0.17	0.05	0.04	0.11	0.00
In general, I think breastfeeding a 2-year-old child would benefit a child’s physical health.		**0.81**	0.16	0.09	0.07	0.26	0.02
In general, I think breastfeeding a child of 3 years or older would benefit a mother’s physical health.		**0.80**	0.14	0.12	0.11	0.11	0.19
In general, I think breastfeeding a 2-year-old child would benefit a mother’s physical health.		**0.68**	0.13	0.16	0.13	0.35	0.26
I think that it is acceptable for 3-year-old or older children to be breastfed by their mothers.		**0.55**	0.50	0.17	0.33	0.15	−0.11
Attitudes Subscale 2: Encouragement to stop breastfeeding	0.81						
I would encourage a woman breastfeeding her 2-year-old child to wean.		0.23	**0.86**	0.07	0.14	0.03	0.09
I would encourage a woman breastfeeding her 3-year-old or older child to wean.		0.34	**0.77**	0.09	0.14	0.11	0.10
I would encourage a woman breastfeeding her 1-year-old child to wean.		−0.03	**0.74**	−0.03	−0.15	0.17	0.17
I think that it is acceptable for 2-year-old children to be breastfed by their mothers.		0.31	**0.63**	0.19	0.42	0.24	−0.01
Attitudes Subscale 3: Mother’s psychological wellbeing	0.91						
In general, I think breastfeeding a 2-year-old child could cause psychological harm to the mother.		0.09	0.13	**0.94**	0.06	0.06	0.13
In general, I think breastfeeding a 1-year-old child could cause psychological harm to the mother.		0.00	0.05	**0.93**	−0.03	0.07	0.10
In general, I think breastfeeding a child of 3 years or older could cause psychological harm to the mother.		0.31	0.02	**0.84**	0.08	0.05	0.15
Attitudes Subscale 4: Embarrassment related to public breastfeeding and its social acceptability	0.71						
I would be embarrassed if a mother breastfed her 1-year-old child in front of me.		−0.08	−0.18	−0.02	**0.71**	0.06	0.13
I would be embarrassed if a mother breastfed her 2-year-old child in front of me.		0.22	0.38	0.01	**0.70**	0.14	0.10
I would be embarrassed if a mother breastfed her child of 3 years or older in front of me.		0.39	0.29	0.15	**0.61**	−0.02	−0.09
Attitudes Subscale 5: Breastfeeding a 1-year-old child	0.70						
In general, I think breastfeeding a 1-year-old child would benefit a child’s physical health.		0.46	0.11	0.06	−0.13	**0.70**	0.08
I think that breastfeeding a child beyond 1 year of age is normal.		0.20	0.24	0.02	0.41	**0.64**	−0.01
I think that it is acceptable for 1-year-old children to be breastfed by their mothers.		0.02	0.39	0.05	0.42	**0.58**	−0.05
In general, I think breastfeeding a 1-year-old child would benefit a mother’s physical health.		0.37	−0.00	0.32	−0.03	**0.55**	0.41
Attitudes Subscale 6: Psychological wellbeing of a child	0.68						
In general, I think breastfeeding a 1-year-old child could cause psychological harm to the child.		0.00	0.01	0.10	−0.02	0.08	**0.83**
In general, I think breastfeeding a 2-year-old child could cause psychological harm to the child.		0.25	0.32	0.26	0.19	0.06	**0.70**
In general, I think breastfeeding a child of 3 years or older could cause psychological harm to the child.		0.52	0.29	0.21	0.18	−0.15	**0.50**

*Note.* The extraction method was exploratory factor analysis with varimax rotation. Factor loadings forming the factors are in bold.

### Distribution of Knowledge and Skills Items

The median knowledge and skills level of the respondents (*N* = 197) was 7.0 (Q1–Q3 = 6.0–8.0, range 1–9). The questions were mostly easy for the respondents, but three questions stood out as being more difficult than the others. These were the number of breastfed children approaching 1 year of age in Finland, the financial benefits of breastfeeding, and the need to stop nighttime breastfeeding after the child turns 1 year old. The distribution of the knowledge and skills item answers is described in Table 4.

**Table 4.** Distribution of Answers in the Knowledge and Skills Items (*N* = 197).

Knowledge and Skills Item (Correct Answer)	Correct Answer	Incorrect Answer
	*n* (%)	*n* (%)
In Finland, there are _____ children approaching the age of 1 who are breastfed (58%)	30 (15.2)	167 (84.8)
The mother should stop breastfeeding the older child after becoming pregnant (No)	188 (95.4)	9 (4.6)
The mother should stop nighttime breastfeeding when the child reaches 1 year of age (No)	118 (60.0)	79 (40.0)
In Finland, it is recommended to stop breastfeeding when the child reaches 1 year of age (No)	184 (93.4)	13 (6.7)
Breastfeeding beyond 12 months is financially beneficial for the family (Yes)	77 (39.1)	120 (60.9)
Breast milk is good nutrition for a child over 1 year old (Yes)	164 (83.2)	33 (16.8)
Women who have breastfed for more than a year have a ____ risk of premenopausal breast cancer (Lower)	175 (88.8)	22 (11.2)
Children breastfed for more than a year have a ____ risk of childhood and adult obesity (Lower)	167 (84.8)	30 (15.2)
The effect of breastfeeding beyond 12 months on the attachment relationship between parent and child is (Positive)	170 (86.3)	27 (13.7)

### Distribution of Attitude Items

The median attitude level of the respondents (*n* = 190) was 4.09 (Q1–Q3 = 3.53–4.69, range 1–5, as 1 = *most negative attitude*; 5 = *most positive attitude*). Most of the respondents expressed positive attitudes towards breastfeeding a 1-year-old child, but the negative and neutral attitudes increased as the breastfed child grew older. The distribution of the responses to the attitude items is described in Table 5.

**Table 5.** Distribution of Public Health Nurses’ Responses to Attitude Items (*N* = 190).

Attitude Item	Agree	Somewhat Agree	Neither Agree or Disagree	Somewhat Disagree	Disagree	Missing
	*n* (%)	*n* (%)	*n* (%)	*n* (%)	*n* (%)	*n* (%)
I think that breastfeeding a child beyond 1 year of age is normal.	138 (72.6)	46 (24.2)	- (-)	6 (3.2)	- (-)	- (-)
I think that it is acceptable for a child to be breastfed.						
1-year old child	179 (94.2)	11 (5.8)	- (-)	- (-)	- (-)	- (-)
2-year-old child	133 (70.0)	37 (19.5)	6 (3.2)	11 (5.8)	3 (1.6)	- (-)
3-year-old or older child	74 (38.9)	57 (30.3)	17 (8.9)	31 (16.3)	11 (5.8)	- (-)
I would be embarrassed if a mother breastfed her child in front of me.						
1-year old child	- (-)	- (-)	- (-)	2 (1.1)	187 (98.4)	1 (0.5)
2-year-old child	2 (1.1)	11 (5.8)	6 (3.2)	22 (11.6)	149 (78.4)	- (-)
3-year-old or older child	9 (4.7)	34 (17.9)	14 (7.4)	44 (23.2)	89 (46.8)	- (-)
I would encourage a woman breastfeeding her child to stop breastfeeding.						
1-year old child	1 (0.5)	2 (1.1)	2 (1.1)	8 (4.2)	176 (92.6)	1 (0.5)
2-year-old child	4 (2.1)	11 (5.8)	11 (5.8)	22 (11.6)	141 (74.2)	1 (0.5)
3-year-old or older child	11 (5.8)	16 (8.4)	14 (7.4)	31 (16.3)	118 (62.1)	- (-)
I think breastfeeding would benefit a child’s physical health.						
1-year old child	129 (67.9)	41 (21.6)	14 (7.4)	3 (1.6)	2 (1.1)	1 (0.5)
2-year-old child	67 (35.3)	33 (17.4)	45 (23.7)	34 (17.9)	10 (5.3)	1 (0.5)
3-year-old or older child	50 (26.3)	28 (14.7)	52 (27.4)	34 (17.9)	25 (13.2)	1 (0.5)
I think breastfeeding could cause psychological harm to the child.						
1-year old child	1 (0.5)	- (-)	2 (1.1)	2 (1.1)	179 (94.2)	6 (3.2)
2-year-old child	2 (1.1)	3 (1.6)	7 (3.7)	19 (10.0)	156 (82.1)	3 (1.6)
3-year-old or older child	5 (2.6)	8 (4.2)	26 (13.7)	44 (23.2)	104 (54.7)	3 (1.6)
I think breastfeeding would benefit a mother’s physical health.						
1-year old child	148 (77.9)	24 (12.6)	10 (5.3)	3 (1.6)	2 (1.1)	3 (1.6)
2-year-old child	74 (38.9)	40 (21.1)	51 (26.8)	14 (7.4)	9 (4.7)	2 (1.1)
3-year-old or older child	52 (27.4)	30 (15.8)	72 (37.9)	20 (10.5)	13 (6.8)	3 (1.6)
I think breastfeeding could cause psychological harm to the mother.						
1-year old child	4 (2.1)	13 (6.8)	11 (5.8)	33 (17.4)	127 (66.8)	2 (1.1)
2-year-old child	3 (1.6)	14 (7.4)	25 (13.2)	29 (15.3)	117 (61.6)	2 (1.1)
3-year-old or older child	6 (3.2)	19 (10.0)	38 (20.0)	38 (20.0)	87 (45.8)	2 (1.1)

## Discussion

This study developed and piloted the Long-Term Breastfeeding Competence Scale (LBCS) which can be used to examine health professionals’ knowledge, skills, and attitudes regarding breastfeeding beyond 12 months. The pilot study indicated preliminary conceptual validity and reliability based on internal consistency. In addition, the expert panel assessments supported the construct validity of the instrument.

Although the internal consistency of the entire instrument was at a good level, Cronbach’s alpha and Rasch analysis did not support the internal consistency of the knowledge and skills dimension alone. Based on previous studies, it can be assumed that the respondent’s attitude towards long-term breastfeeding is also reflected in the responses to the knowledge items (Baranowska et al., 2019; Cockerham-Colas et al., 2012; Colaceci et al., 2020; Goldbort et al., 2023; Radaelli et al., 2012; Rempel & McCleary, 2012; Zhuang et al., 2020). This study also found that the knowledge and skills instrument could not be validated on its own because the affect of the attitude could be seen in the knowledge and skills level.

Among the areas of competence, skills are not included in the instrument as desired, but knowledge and attitudes dominate instead. When complementing the original instrument through a systematic review, a gap in knowledge was found regarding counseling skills related to breastfeeding beyond 12 months. Therefore, the instrument does not specifically reach the public health nurses’ practical know-how regarding counseling situations, but instead describes the relevant counseling contents. Breastfeeding counseling skills can be considered to manifest as a capacity to apply knowledge effectively in various situations through observation and communication (Mulcahy et al., 2022). Due to this, knowledge and skills regarding breastfeeding counseling can be perceived as an entity, which is also visible in previous studies (Sandhi et al., 2023).

As the previously used instruments (Baranowska et al., 2019; Cockerham-Colas et al., 2012; Zhuang et al., 2020) focused predominantly on attitudes with the knowledge dimension remaining narrow, the aim of the present study was to be able to measure competence more broadly. As the LBCS measures knowledge more broadly, it may identify connections between knowledge, skills and attitudes. By identifying knowledge gaps, it is possible to target breastfeeding education according to needs, and thus influence the negative attitudes that constitute a barrier to evidence-based breastfeeding counseling.

Although the target population in this pilot study was public health nurses, previous studies have shown that there are also negative attitudes and knowledge gaps related to long-term breastfeeding in other professions, for example pediatrics (Baranowska et al., 2019; Cockerham-Colas et al., 2012; Radaelli et al., 2012). The LBCS can also be used to examine the competence of other professional groups because the items are not restricted to the maternity and child health clinic context. In the future, it would be valuable to study different professions as, in Finland, no breastfeeding education is included in physicians’ training. Mothers breastfeeding their child beyond infancy may encounter several different professional groups in healthcare settings, and the competence levels of health professionals play a role in how reliable mothers perceive the service in general (Säilävaara, 2023).

### Limitations

Long-term breastfeeding is a topic that divides opinions more than breastfeeding during infancy. A sensitive topic can affect the sample in an online survey, in which the respondents can choose to participate. People may favor a survey based on their personal interests, which can cause bias in the results.

As the aim of this study was to develop and validate a new scale, it is to be considered that the results regarding competence are preliminary findings of the pilot study and also that reliability was assessed only from the point of view of internal consistency. More research is needed to obtain more comprehensive information on reliability and the psychometric properties of the scale.

The LBCS was validated in the Finnish healthcare context and the distribution of the items can be seen to reflect a breastfeeding-friendly Nordic country. This weakens the generalizability of the results, as breastfeeding cultures vary in developed countries. If using the LBCS in another context, its validity must be reassessed.

## Conclusions

The LBCS is an applicable instrument that can be used to examine public health nurses’ competence regarding breastfeeding beyond 12 months and it may also be used with other professional groups and different kinds of breastfeeding supporters, for example volunteer workers. This study, which examined competence in its entirety, found that the attitudes of public health nurses are also reflected in their knowledge and skills. In conclusion, this highlights the importance of influencing health professionals’ attitudes.

## Supplemental Material

sj-docx-1-jhl-10.1177_08903344241254343 – Supplemental material for Developing an Instrument to Measure Public Health Nurses’ Competence Related to Breastfeeding Beyond 12 Months Supplemental material, sj-docx-1-jhl-10.1177_08903344241254343 for Developing an Instrument to Measure Public Health Nurses’ Competence Related to Breastfeeding Beyond 12 Months by Niina Pöyhönen, Oona Ojantausta, Marja Kaunonen, Katri Vehviläinen-Julkunen and Riikka Ikonen in Journal of Human Lactation
